# Modern Medico-Psychology and Psychiatry

**Published:** 1894-04-14

**Authors:** T. S. Clouston

**Affiliations:** Lecturer on Mental Diseases, Edinburgh University, and Physician Superintendent Royal Asylum, Morningside


### Modern Medico-Psychology and Psychiatry.

By T. S. Clouston, M.D., Lecturer on Mental Diseases, Edinburgh University, and Physician Superintendent Royal Asylum, Morningside.

### III.—LEGISLATIVE AND STATE MEASURES AND THEIR EFFECTS—(*continued*).

One of the questions the Commissioners have asked in all asylums has been "What is the state of the worst class of patients?" "Is their degeneration of habits allowed to go on, or are strenuous attempts made to arrest it?" Irritability and want of inhibition is part of their disease, and therefore the Scripture precept, of a soft answer turning away wrath, is worth countless bottles of sedative medicine. A good meal, and a smoke of tobacco, a few hours hard work in the garden, a dance and a warm bath, a good bed and a kindly attendant, were universal prescriptions. All this common sense philosophy and psychology the Commissioners have gone about preaching and trying to enforce for forty-eight years. The æsthetics of life, bright colours, pictures, singing birds and flowers they did not neglect. They did not lightly pass over offences and breaches of the law. It is a serious offence to neglect or to strike, or to ill-use a patient by an attendant or nurse under any circumstances whatever, and the Commissioners never condone such offences when they come to their knowledge but insist on prosecution. It is an offence to keep any insane person for profit uncertified, and they invariably order a prosecution in such cases, so as to carry out universally the intention of the law that all the mentally unsound shall be under authorised care and inspection. They have mostly been ready to take up improvements in asylum management and the general treatment of insanity from abroad and promulgate them. They have never looked on new modes of treatment by drugs nor on scientific or pathological investigation as being specially within their province. In this I think they have taken too narrow a view of their duty.

One most unfortunate tendency has strongly developed itself of late years in England. Whether the Commission is chiefly to blame, or the local committees of visitors of asylums, it is difficult to say. This is the building of new asylums for over 500 patients, and the constant enlargement of the county asylums by additions and annexes, so that many of them have grown utterly unwieldly, some of them now containing over 2,000, the patients being treated in masses and not individually. Some of the single wards of these gigantic structures often consist of monstrous aggregations of sometimes over 100 patients. The temptation was great when an asylum of say 500 patients became too small, to add 100 more and then another 100. But in such great counties as Lancashire and Yorkshire, with many towns of 20,000 inhabitants and over, the policy of planting a new asylum of moderate size near each town, should certainly have been adopted as new accommodation was required. Instead of that, vast buildings, each containing many hundreds of patients, were erected in connection with the existing asylums; so that now there is scarcely a proper sized institution in Lancashire or Yorkshire, or the metropolitan counties. This has been the one grand blunder of English lunacy administration, and I fear it is now almost irremediable in some instances. Could my words in these articles have any effect in convincing the local authorities of England that this tendency to vast aggregations of insane people in the same institution is bad for the insane, bad for their relatives, bad for the public, bad for the asylum doctors and staff, and bad for the ratepayers, they will not have been written in vain. The system is unmedical and unscientific in the highest degree. It defeats to a large extent the humane intentions of the original author of the lunacy laws. It takes those suffering from a disease, the strongest tendency of which is a diminution of the social faculties of man, and it places them under conditions where this morbid tendency must necessarily be greatly strengthened instead of being actively counteracted. The relatives of patients, who by visits can do much in most cases to keep them social and human, cannot visit them because the asylums are too far away, and soon forget and neglect them. The curable and the incurable tend to be subjected to the same routine. A patient labouring under many forms of recent insanity needs intimate study of his individual symptoms, mental and bodily. How can this be given to each in an institution with 2,000 patients? The incurable must be treated in squads in such an institution, and this method will necessarily infect the minds of the staff and be applied to the curable also. It will become by a psychological law the "system" of the place. Had the Commissioners obeyed their medical instincts they would, from the first, have sternly fought against monster asylums; they would have taken pains to educate the public and the Government on this matter, and they would have urged every town that had 200 insane to build an asylum for itself. The medical profession and the country would have backed them up in this policy. In the name of humanity and medicine let this policy go no further. I have lived among the insane for over thirty years, but a visit to one of the new English "Annexes" for 500 patients, with its great wards of 100 incurable patients in each, positively makes me depressed and hopeless in my work. For the same money charming little hospital asylums of from 200 to 400 inmates each could have been put up in the districts near where the patients lived. I am not prepared to say that for certain classes of the quiet, friendless, chronic insane, a very few institutions for 1,000 patients may not be suitable in some counties; but if small asylums are not better for cure, for comfort, and for efficiency all round, my medical experience has gone for nothing.

The Commission and the laws it had to administer have undoubtedly given satisfaction to the country generally. But in and about London there have always been a sufficient number of "cranks," of half-cured lunatics let out of asylums after the first intensity of their mental attack has passed off, of borderland cases trembling for their shaky wits, to whom the name of asylum is anathema, to keep up an agitation against asylums, Commissioners, doctors, and existing lunacy laws. Some few unfortunate infractions of the Statute, and lunacy law suits in consequence, undoubtedly produced an impression on the minds of some of the public that the law required revision. In 1877 therefore the House of Commons, at the initiative of Mr. Dilwyn, appointed a Committee to take evidence and frame a report on the subject. It examined most of those experienced in the working of the lunacy laws in the kingdom; it allowed all who alleged grievances under the action of these laws to come before it and give evidence, and it drew up a report recommending certain changes in the laws but it concluded that no effective evidence of the detention of really sane persons as lunatics had been laid before it.

(*To be continued.*)

